# Fetal Bovine Serum RNA Interferes with the Cell Culture derived Extracellular RNA

**DOI:** 10.1038/srep31175

**Published:** 2016-08-09

**Authors:** Zhiyun Wei, Arsen O. Batagov, David R. F. Carter, Anna M. Krichevsky

**Affiliations:** 1 Department of Neurology, Brigham and Women’s Hospital and Harvard Medical School, Boston, Massachusetts 02115, USA; 2 Genome and Gene Expression Data Analysis Division, Bioinformatics Institute, Singapore 138671, Singapore; 3 Department of Biological and Medical Sciences, Faculty of Health and Life Sciences, Oxford Brookes University, Oxford, OX3 0BP, UK

## Abstract

Fetal bovine serum (FBS) has been used in eukaryotic cell cultures for decades. However, little attention has been paid to the biological effects associated with RNA content of FBS on cell cultures. Here, using RNA sequencing, we demonstrate that FBS contains a diverse repertoire of protein-coding and regulatory RNA species, including mRNA, miRNA, rRNA, and snoRNA. The majority of them (>70%) are retained even after extended ultracentrifugation in the preparations of vesicle-depleted FBS (vdFBS) commonly utilized in the studies of extracellular vesicles (EV) and intercellular communication. FBS-associated RNA is co-isolated with cell-culture derived extracellular RNA (exRNA) and interferes with the downstream RNA analysis. Many evolutionally conserved FBS-derived RNA species can be falsely annotated as human or mouse transcripts. Notably, specific miRNAs abundant in FBS, such as miR-122, miR-451a and miR-1246, have been previously reported as enriched in cell-culture derived EVs, possibly due to the confounding effect of the FBS. Analysis of publically available exRNA datasets supports the notion of FBS contamination. Furthermore, FBS transcripts can be taken up by cultured cells and affect the results of highly sensitive gene expression profiling technologies. Therefore, precautions for experimental design are warranted to minimize the interference and misinterpretations caused by FBS-derived RNA.

Deep sequence analysis of extracellular RNA (exRNA) released by cultured cells in forms of extracellular vesicles (EVs) and lipoprotein complexes (RNPs) is an expanding area of research. An important question raised years ago and still highly debated in the field, is whether specific RNA species or motifs are enriched in EVs and RNPs. Such enrichment would support an active and regulated mechanism of RNA secretion. Alternatively, exRNA may reflect the entire cellular transcriptome, a specific RNA class (e.g. miRNA), or one of the steps of RNA metabolism, and thus, provide a peripheral measure for monitoring the cellular transcriptome. Addressing this question is critical for the study of intercellular communication and development of RNA biomarkers. Cell culture systems employed in the studies of exRNA biogenesis and release, rely heavily on the fetal bovine serum (FBS), a commonly used reagent vital for the growth of numerous cell types. Although many reports characterized exRNA isolated from human and mouse serum and suggested serum RNA as the major source of disease biomarkers^1^, little attention has been paid to confounding effects of FBS-derived RNA on cell-culture based studies. Here, we characterize the RNA content of FBS and vesicle-depleted FBS (vdFBS), and demonstrate its interference with cell-derived exRNA isolated from conditioned media and, possibly, cellular RNA.

## Results and Discussion

### Enrichment of specific miRNAs in cell-derived exRNA is likely caused by FBS-derived RNA contamination.

**Figure 1:**
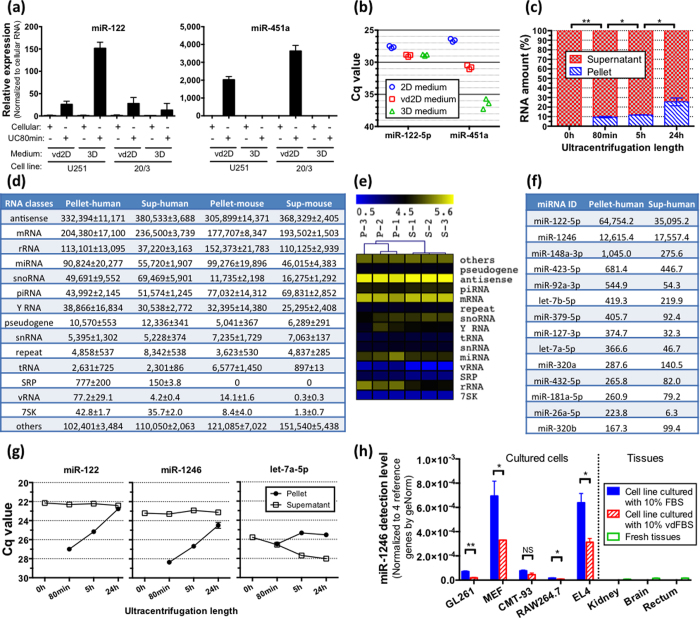
FBS-derived RNA interferes with RNA analysis of exRNA and cellular RNA. **(a)** qRT-PCR analysis of 20/3 and U251 glioma cellular and EV-associated exRNA demonstrates that miR-122 and miR-451a are highly enriched in EVs when the cells are cultured with FBS (2D media). miR-451a is not detected in exRNA of cells cultured in the serum-free 3D medium. N = 3 cells per group. **(b)** miR-122 and miR-451a levels were determined in fresh media, including 2D medium containing 10% FBS, vd2D medium containing 10% FBS pre-spun for 24 h, and serum-free 3D medium. Low Cq values correspond to high levels of the miRNA. N = 3 media aliquots. **(c)** Percent of total RNA isolated from FBS pelleted and supernatant fractions after UC for 80 min, 5 hr, or 24 hr. N = 3 FBS aliquots per group with two-tailed t-test. **(d)** RNA-seq analysis shows RNA composition of FBS pellets or supernatants (24 hr UC). The reads were mapped to either human or mouse genomes, and the data is shown as mean ± SEM RPM. N = 3 FBS aliquots. **(e)** Pearson clustering analysis of logarithm-transformed data based on major RNA composition shows separation of three supernatant samples (denoted as S) from three pellet samples (denoted as P). **(f)** The most abundant miRNAs in FBS pellet and supernatant fractions (24 h UC), as detected by RNA-seq. Of note, miR-451 was not sequenced from the NEBNext small RNA libraries. **(g)** The levels of selected miRNAs in FBS pellets and supernatants were determined by qRT-PCR. N = 3 FBS aliquots per group. **(h)** Bovine and primate-specific miR-1246 is detected by qRT-PCR in mouse cultured cells but not mouse tissues. N = 3 cells or tissue slices per group. *p < 0.05; **p < 0.01; NS, not significant. All bars represent mean ± SEM in the figure.

Several observations suggested that FBS-associated RNA might interfere with the analysis of exRNA. First, we detected high levels of miR-122, a specific miRNA abundantly expressed in liver but undetected in other tissues^2^,^3^, in the conditioned media of glioma cell lines cultured as a monolayer or neurospheres (Fig. 1a). Considering the lack of miR-122 expression in glioblastoma tumors^4^ and cultured glioma cells (Fig. 1a), we rationalized that this finding could be explained by either unusually efficient miR-122 secretion, or more plausibly, culture media as a primary source of this miRNA. Indeed, miR-122 was highly abundant in fresh unconditioned media utilized for glioma cell cultures (Fig. 1b), indicating that the majority of miR-122 in the conditioned medium comes from the medium components rather than cell secretion. Another specific microRNA, miR-451a, expressed in diverse cells and tissues with highest abundance in the blood^2^, was reported as the most enriched miRNA in EVs secreted by various cell types^5^,^6^,^7^,^8^,^9^,^10^,^11^. Consistent with these findings, we found miR-451a enriched in the pelleted EVs isolated from the conditioned media of U251 and 20/3 glioma monolayer cultures grown in DMEM/10% vdFBS (Fig. 1a). However, when the same cell lines were cultured at the same glucose concentration but in serum-free neurosphere-promoting conditions, miR-451a was not detected in the ultracentrifugation (UC) pellets/exRNA fraction (Fig. 1a). To better understand the contribution of different culture media, we isolated total exRNAs from fresh unconditioned “monolayer culture medium” (2D medium, containing DMEM/10% FBS), vesicle-depleted 2D medium (vd2D, containing FBS after 24 h UC), and “neurosphere medium” (3D medium, serum-free), and measured miR-451a levels using qRT-PCR. MiR-451a was abundant in the 2D medium, slightly reduced in the vd2D medium, and barely detected in the 3D medium (Fig. 1b). Of note, the levels of miR-122 and miR-451 in the vd2D media relative to the crude 2D media were reduced to a different extent, but not entirely abolished. Although the possibility that culture conditions affect RNA secretion cannot be ruled out, these findings suggest that UC-based vesicle depletion does not fully eliminate RNA from the FBS/culture media, and that FBS-associated RNA may lead to false results and interpretation such as the previously reported miR-451a enrichment in exRNA.

### RNA cannot be depleted from FBS using ultracentrifugation

We next set to characterize the composition of FBS RNA and investigate whether RNA can be removed from serum. Serum RNA is associated with exosomes and other EVs, as well as non-membrane lipid and protein RNPs. Most researchers use 70,000 g to 110,000 g UC for 2 h–18 h to produce vdFBS. It has been demonstrated that prolonged UC removes the majority, although not all EVs, as monitored by NanoSight^12^. However, to what extend the vdFBS is RNA-depleted is unclear. To address this question, we spun FBS samples at 100,000 g for 80 min, 5 h, or 24 h and isolated total RNA from the pelleted (i.e. EV-enriched) material and the supernatants (i.e. EV-depleted). RNA was also isolated from crude FBS at the baseline. 5–40 ng/ml total RNA was isolated from the crude FBS, varying between the scales and batches, and consistent with the previous assessments of human serum^13^. As shown in Fig. 1c, even the prolonged 24-hour UC extended beyond the reported protocols, removed only a part of the RNA (19–33%) from FBS (Fig. 1c). On average, the vdFBS preparations contained 34 ng/ml RNA, the majority of which was most likely associated with non-vesicular complexes, such as RNPs. Regardless of the source of this RNA (vesicular or not), it can potentially contaminate cell culture condition media and co-isolate/precipitate with the cell culture EVs in the subsequent procedures.

### FBS contains a diverse repertoire of conserved RNA species indistinguishable from human and mouse transcripts

To further evaluate the potential of FBS RNA to interfere with cell culture derived exRNA, we performed RNA deep sequencing of the FBS fractions. Three different batches of FBS were spun at 100,000 g for 24 h, and the RNA samples isolated from the pelleted EV-enriched fractions and EV-depleted supernatants were used for the construction of the NEBNext small RNA libraries, followed by the HiSeq 2000 RNA-seq. Read mapping to the hg19 version of Human Genome was performed using the exceRpt small RNA-seq pipeline V2.2.8 of Genboree Workbench with default parameters^14^. On average, 13.6% and 21.7% reads from the “pellet RNA” and “supernatant RNA” fractions, respectively, were mapped to the human genome, suggesting that a significant part of FBS-associated transcripts is evolutionally conserved and could contribute to false-positives in the analysis of human cells. With precise no-mismatch-only mapping allowed, 9.2% and 13.2% reads of the respective fractions were mapped to the human genome. Major RNA composition of the two fractions, represented as reads per million mapped (RPM), is shown in Fig. 1d. Notably, both EV-enriched and EV-depleted fractions contained diverse RNA species, including mRNA, rRNA, miRNA, and other non-coding transcripts (antisense, snoRNA, Y RNA, etc.). The differences between the two sets of fractions were largely quantitative, with miRNA and rRNA fragments relatively enriched in the pelleted fractions, and mRNA and snoRNA – in the supernatants. Based on the RNA repertoire, Pearson clustering analysis demonstrated a clear separation between EV-enriched and EV-depleted fractions (Fig. 1e).

From 5.2% to 12.9% reads mapped to human genome corresponded to miRNA. MiR-122 was the most abundant miRNA in the FBS, followed by miR-1246, miR-423-5p, miR-148a-3p, and let-7 family (Fig. 1f). High levels of these miRNAs in the FBS were further confirmed by qRT-PCR (Fig. 1g). The depletion efficiencies, defined as abundance ratios between the pellet and supernatant fractions, were substantially different for these miRNAs, suggesting their distinct association with EVs and RNPs. Of note, all miRNAs mentioned above have been previously reported as enriched in cell culture-derived exRNA relative to cellular RNA^8^,^10^,^15^,^16^. It might be worth revisiting those results as they do not take FBS RNA into account and almost certainly overestimate secretion and enrichment of specific exRNA species in the conditioned media. FBS RNA reads were also mapped to mouse genome (Fig. 1f), indicating that FBS RNA could affect the results of both human and rodent culture exRNA analysis. Therefore, despite low levels of FBS RNA, its contribution should be carefully considered in the exRNA research, as misannotated bovine transcripts will lead to false positives and misinterpretations, especially when the determinants of RNA release and enrichment in extracellular complexes are investigated.

### FBS-derived miR-1246 is detected in cultured mouse cells

The additional question of broader importance is whether FBS-associated RNA may interfere with cellular RNA analysis. MiR-1246 gene that encodes one of the most abundantly expressed miRNAs in FBS, is present in only 4 out of 223 species, including bovine, human, orangutan and chimpanzee, but not in mouse or rat^17^. There are no sequences homologous to hsa-miR-1246 identified in the mouse genome. However, low levels of mature miR-1246 were consistently and reproducibly detected in all tested mouse cell lines cultured with 10% FBS (Fig. 1h) by both LNA-based SYBR Green assay (Exiqon) and TaqMan assay (Thermo Fisher Scientific). miR-1246 signal varied between the recipient cells lines, suggesting different levels of uptake and/or processing in the different cells. The miR-1246 signal was significantly reduced in cells that were switched to 10% vdFBS culture medium seven days prior to RNA isolation and undetectable in mouse tissues (Fig. 1h). This example suggests that FBS RNA complexes associated with EVs and possibly non-vesicular RNPs as well, might be taken up by cultured cells and interfere with the quantification of cellular RNA. Considering highly sensitive expression profiling technologies commonly utilized in current research that are capable of detecting femtomoles of RNA, further in-depth analysis of FBS RNA-associated confounders is warranted. As of today, there are no data suggesting the functional activity of this low-level bovine RNA in the recipient cells; nevertheless, subsequent work will be required to accurately address its impact.

### Bovine-specific reads are found in public cell culture exRNA datasets

To further confirm that FBS-derived RNA contamination is common in cell culture exRNA, we interrogated several public exRNA-seq datasets and examined whether they contain bovine-specific reads. Totally, 13 exosomal RNA samples and 8 corresponding human cellular RNA samples from 4 different cell types were analyzed. To adjust variable mapping rates between the samples, the ratio of bovine-specific reads to the human-specific reads was used as the normalized level for bovine contamination. In the exosomes, roughly 2.6–17.2% of reads (the inter-quartile range) corresponded to bovine-specific transcripts. As shown in Table 1, bovine-specific reads were detected in both cellular RNA and exRNA, and exRNA contained more bovine contamination than corresponding cellular RNA. Although statistical test is not meaningful within each individual dataset, limited by small sample sizes, we performed a nonparametric Mann–Whitney *U* test for all the samples together as a global comparison. Bovine-contamination was significantly more severe in exRNA than in cellular RNA (exact p = 0.016). Therefore, the analysis of public datasets is indicative of common contamination of FBS-derived RNA in the various exRNA samples preparations.

### Further considerations related to FBS/media effects on cell culture based studies

This data lead us to consider several factors that influence cell culture-based exRNA research. First, media composition, including serum, growth factors, and other natural derivatives, would affect the results of exRNA analysis. As demonstrated in Fig. 1a, the same cells cultured in various media exhibit distinct exRNA profiles and “enrichment” of different exRNA species that are, in fact, caused by media contaminants. Second, FBS/media pre-processing procedures have differential effects on RNA depletion. For example, as demonstrated in Fig. 1c, the prolonged UC pellets some miRNAs more efficiently than others. Different rotor types further contribute to the variability^18^. Third, even under identical experimental conditions, FBS and media brands and batches represent additional confounding factors.

Several controls should be considered to help minimize these factors. First, analysis of cell culture-derived exRNA should include RNA samples isolated using the same procedure from the corresponding fresh media. Of note, various cell types and lines secrete vastly different amounts of exRNA ranging from a few nanograms to a hundred nanograms per ml of conditioned media^8^,^19^. For example, the amount of EV RNA we isolate from heterogeneous glioblastoma patient-derived neurospheres, ranges from 2.3 ng/mL to 25 ng/mL. In some cases, therefore, the anticipated contribution of vdFBS-containing media would be negligible, whereas in others, it may skew the results significantly. Second, a study of intercellular communication and EV effects on recipient cells should include the corresponding EV fractions isolated from the fresh media as a negative control. Such media-derived fractions can cause significant physiological effects in the recipient cells, including altered viability and migration^12^,^20^ that might be partly due to the RNA uptake. Intracellular stability of the internalized RNA-containing FBS/media-derived complexes and their potential activity remains to be investigated. In addition, development of an alternative RNA-free complete media for exRNA research is warranted.

## Methods

### Cell Culture

U251 and 20/3 human glioma cells, GL261 mouse glioma cells, mouse embryonic fibroblast cells (MEF; kind gift from Dr. Dan Xia, Brigham and Women’s Hospital), CMT-93 mouse rectum cells, RAW264.7 mouse macrophage (both were kind gifts from Dr. Shirong Liu, Brigham and Women’s Hospital) and EL4 mouse T lymphocytes (kind gift from Dr. Zuojia Chen, Brigham and Women’s Hospital) were cultured in DMEM (Corning, NY) with 10% FBS (Gibco, MA), either EV-depleted or not, and passaged by trypsinization (Gibco). Glioma cells cultured as neurospheres were grown in Neurobasal medium (Gibco) supplemented with 3 mM GlutaMAX (Gibco), 1x B-27 supplement (Gibco), 0.5x N-2 supplement (Gibco), 20 ng/mL EGF (R&D Systems, MN), and 20 ng/mL FGF (PEPROTECH, NJ). None of these cell lines is in the database for misidentified cell lines (ICLAC). All of them are mycoplasma-free.

### RNA isolation

For cellular RNA isolation, cells were washed thoroughly with cold DPBS (Corning) three times. RNA was then isolated using miRCURY RNA Isolation Kit (Exiqon, Denmark), with on-column DNase (Qiagen, Germany) treatment step added to the manufacturer’s protocol. For tissue RNA isolation, fresh tissues were excised from one C57BL/6J mouse and transferred to Lysis Solution immediately, followed by homogenization. The lysate was spun at 14,000 g for 2 min to remove cell debris, and RNA isolated as above. For exRNA isolation from FBS, either 11.5 mL or 40 mL of freshly thawed FBS were injected into Quick-Seal Centrifuge Tubes and spun at 100,000 g, 4 °C using an Optima L-90K ultracentrifuge (Beckman Coulter, CA) in SW 41 Ti Rotor (28,500 rpm) or SW 28 rotor (28,000 rpm), respectively. RNA from the pellet was isolated as above. Either 2 mL or 20 mL of the supernatant was concentrated using 3 kDa Amicon Ultra Centrifugal Filters (EMD Millipore, MA) at 4,000 g 4 °C for 80 min. The concentrate was then lysed using six volumes of Lysis Solution of miRCURY RNA Isolation Kit (Exiqon), followed by the RNA isolation as above. For exRNA isolation from conditioned or fresh media, 40 mL media was first spun at 300 g 4 °C for 10 min, and further at 2,000 g 4 °C for 15 min, and the supernatants filtered through a 0.8 μm filter (EMD Millipore, MA) to remove cells and cell debris. After adding 10 U/mL SUPERase-In RNase Inhibitor (Thermo Fisher Scientific), EVs were pelleted by ultracentrifugation at 100,000 g 4 °C for 80 min in an SW 28 rotor, and RNA isolated from EV pellets as described above. The concentration of cellular/tissue RNA was determined using NanoDrop 2000 Spectrophotometer (Thermo Fisher Scientific), and concentration of exRNA was determined using Quant-iT RiboGreen RNA Assay Kit (Thermo Fisher Scientific).

### Quantitative reverse transcriptase-polymerase chain reaction (qRT-PCR)

Ten ng total RNA in 10 μl of reverse transcription reaction mix was used for Universal cDNA Synthesis kit II (Exiqon). The cDNA was diluted 80 times, and 4 μl was used in 10 μl qPCR reactions with ExiLENT SYBR Green and LNA primers (Exiqon). The qPCR reactions were performed on a ViiA 7 instrument (Thermo Fisher Scientific) in triplicates. Alternatively, for amplification of cell/tissue RNA, 100 ng of total RNA was used as input and cDNA was diluted 30 times. The U6 snRNA was monitored as the endogenous control for cellular RNA. A panel of 4 endogenous controls (U6, U2, miR-103a-3p and miR-24-3p) was used for geNorm normalization between cells and tissues. The specificity of all qPCR primers was verified by the melt curve analysis. For qRT-PCR using TaqMan assays, manufacturer’s protocol was carried out with the following modifications: the input of 3.33 ng RNA was used for 5 μl reverse transcription reaction and PCR reactions were performed in 10 μl volume.

### RNA sequencing

60 ng of total RNA was treated with T4-PNK and used as an input for NEBNext small RNA library construction (New England Biolabs, MA), with size selection for 15-65nt inserts. The libraries were examined using Agilent Bioanalyzer 2100 (Agilent, CA), quantified with KAPA Library Quantification Kits for Illumina (KAPA Biosystems, MA), and sequenced on HiSeq 2000 (Illumina, CA) with single-end 50 bp reads, at the Center for Cancer Computational Biology at Dana-Farber Cancer Institute. On average, 19 million reads per sample were produced. Reads annotation to hg19 was performed using exceRpt small RNA-seq pipeline V2.2.8 of the Genboree Workbench^14^. The data was normalized to total mapped reads. The hierarchical clustering analysis and the heat map were produced using MultiExperiment Viewer (Dana-Farber Cancer Institute, MA). Data accompanying this paper has been submitted to GEO under GSE78970.

### Bioinformatical analysis on public RNA-seq datasets

Raw sequence data were downloaded from the NCBI GEO database or SRA database. For the SRA files, they were converted to the FASTQ format using the SRA toolkit v2.6. The quality of the reads was estimated with the FastQC software v0.11. Based on the FastQC report, the optimal read spans were identified as the longest uninterrupted read sequence spans, where for all the base positions the quality scores exceeded 30, on the PHRED scale. The datasets, whose optimal read spans were shorter than 20 bases, were discarded from the analysis. The reads were mapped, using the Bowtie2 software v2.2.8 with the following set of parameters: -p 8, -local, -L 25, -X 1300, -N 0. For each individual FASTQ file containing the sequencing reads of a given experiment, the reads were aligned independently onto two unmasked reference genomes: H.sapiens (GRCh38) and B.taurus (UMD3.1.1). The species-specific reads were defined as such reads that align to only one of the reference genomes. Considering variable aligned reads count between samples/datasets, human-specific reads count was used to normalize bovine-specific reads count. Therefore, the ratio of bovine-specific reads count to human-specific reads count was calculated as the index to quantify FBS-derived RNA contamination. To accelerate the sample analysis, a random subsampling of 1 million reads from each analyzed FASTQ file, using the seqtk software tool, was used as input for the analysis.

### Statistics

When two groups were compared, significance was determined using an unpaired two-tailed t-test. Before performing t-test, normal distribution was verified by 1-sample K-S test and equality of variances was verified by Levene’s test using SPSS (IBM, NY). To assess the statistical significance of difference of bovine-specific reads ratio between the exRNA and the cellular RNA, the two-tailed Mann–Whitney U test was applied using SPSS. A p value < 0.05 is considered as statistical significance. All bars on the graphs represent mean ± SEM.

## Additional Information

**How to cite this article**: Wei, Z. *et al.* Fetal Bovine Serum RNA Interferes with the Cell Culture derived Extracellular RNA. *Sci. Rep.* **6**, 31175; doi: 10.1038/srep31175 (2016).

## Figures and Tables

**Table 1:** Bovine-specific reads are detected in public exRNA-seq datasets.

Bos-specific/Hsa-specifc ratio						
Accession#	Cell type	Cellular RNA	Exosomal RNA	FC	Sample size	Reference
GSE38916	HEK293T	0.029 ± 0.010	0.095 ± 0.026	3.26	2 vs 2	^10^
GSE71901	MCF7	0.0004 ± 0.0004	0.082 ± 0.004	195.4	2 vs 3	^21^
SRP046046	HB	0.063 ± 0.012	0.146 ± 0.040	2.32	3 vs 6	^11^
SRP031761	LIM1863	0.166	0.473 ± 0.169	2.85	1 vs 2	^8^

HEK293T: human embryonic kidney cell line; MCF7: human breast cancer cell line; HB: human B cells; LIM1863: human colon cancer cell line. FC: fold change. Data expressed as mean ± SEM.
